# Single-row vs. double-row arthroscopic rotator cuff repair: clinical and 3 Tesla MR arthrography results

**DOI:** 10.1186/1471-2474-14-43

**Published:** 2013-01-27

**Authors:** Cosimo Tudisco, Salvatore Bisicchia, Eugenio Savarese, Roberto Fiori, Dario A Bartolucci, Salvatore Masala, Giovanni Simonetti

**Affiliations:** 1 Department of Orthopaedic Surgery, University of Rome “Tor Vergata”, 81 Oxford Street, Rome, 00133, Italy; 2 Department of Diagnostic Imaging and Interventional Radiology, Molecular Imaging and Radiotherapy, University of Rome “Tor Vergata”, 81 Oxford Street, Rome, 00133, Italy

**Keywords:** Shoulder, Rotator cuff tear, Arthroscopic repair, MR arthrography, Clinical result

## Abstract

**Background:**

Arthroscopic rotator cuff repair has become popular in the last few years because it avoids large skin incisions and deltoid detachment and dysfunction. Earlier arthroscopic single-row (SR) repair methods achieved only partial restoration of the original footprint of the tendons of the rotator cuff, while double-row (DR) repair methods presented many biomechanical advantages and higher rates of tendon-to-bone healing. However, DR repair failed to demonstrate better clinical results than SR repair in clinical trials. MR imaging at 3 Tesla, especially with intra-articular contrast medium (MRA), showed a better diagnostic performance than 1.5 Tesla in the musculoskeletal setting. The objective of this study was to retrospectively evaluate the clinical and 3 Tesla MRA results in two groups of patients operated on for a medium-sized full-thickness rotator cuff tear with two different techniques.

**Methods:**

The first group consisted of 20 patients operated on with the SR technique; the second group consisted of 20 patients operated on with the DR technique. All patients were evaluated at a minimum of 3 years after surgery. The primary end point was the re-tear rate at 3 Tesla MRA. The secondary end points were the Constant-Murley Scale (CMS), the Simple Shoulder Test (SST) scores, surgical time and implant expense.

**Results:**

The mean follow-up was 40 months in the SR group and 38.9 months in the DR group. The mean postoperative CMS was 70 in the SR group and 68 in the DR group. The mean SST score was 9.4 in the SR group and 10.1 in the DR group. The re-tear rate was 60% in the SR group and 25% in the DR group. Leakage of the contrast medium was observed in all patients.

**Conclusions:**

To the best of our knowledge, this is the first report on 3 Tesla MRA in the evaluation of two different techniques of rotator cuff repair. DR repair resulted in a statistically significant lower re-tear rate, with longer surgical time and higher implant expense, despite no difference in clinical outcomes. We think that leakage of the contrast medium is due to an incomplete tendon-to-bone sealing, which is not a re-tear. This phenomenon could have important medicolegal implications.

Level of evidence III. Treatment study: Case–control study.

## Background

Arthroscopic rotator cuff repair has become popular in the last few years because it avoids large skin incisions and deltoid detachment and dysfunction. It is associated with reduced postoperative pain, and it allows the surgeon to detect and treat other associated shoulder pathologies; moreover it has demonstrated clinical results comparable to open and mini-open repairs [1-3], but some recent studies question the fact of reduced pain after arthroscopy [4,5]. Two different groups of arthroscopic rotator cuff repair techniques are described in literature. Earlier arthroscopic single-row (SR) repair methods achieved only partial restoration of the original footprint of the tendons of the rotator cuff. Subsequently, in several studies, double-row (DR) repair methods showed, when compared to SR repair methods, better fixation strength [6-11], better restoration of the footprint area [7,12-17], less micromovements [18] and greater, but more homogeneous, compression pressure through the tendon [16,17,19]. These biomechanical advantages led to the higher rates of tendon-to-bone healing observed with DR repair in several studies [3,10,20-23]; however, DR repair failed to demonstrate better clinical results than SR repair in clinical trials [10,20,24-27].

MR imaging at 3 Tesla, especially with intra-articular contrast medium (MRA), showed a better diagnostic performance than 1.5 Tesla in the musculoskeletal setting, with a consequent improvement in bone and soft tissue detail [28-38].

The objective of this study was to retrospectively evaluate the clinical and radiological results in two groups of patients operated on for a medium-sized full-thickness rotator cuff tear [39] with either a SR or a DR repair. The primary end point was the re-tear rate at 3 Tesla MR Arthrography (MRA). Magnetic Resonance Arthrography (MRA) was used in order to obtain the best possible visualization of the cuff, as reported by many authors [30,31,33,35]. The secondary end points were the Constant-Murley Scale (CMS) [40], the Simple Shoulder Test (SST) [41] scores, surgical time and implant expense. The null hypothesis was that there were no differences between the two groups.

## Methods

We evaluated clinically and radiologically two groups of patients operated on in our Department by the senior author for an arthroscopic repair of a medium-sized full-thickness rotator cuff tear [39], with either SR repair or “DR double-pulley” repair according to Arrigoni et al. [42].

A review of the literature [3,20-23,25,43-45] revealed that a re-tear rate of approximately 40% for SR repair could be anticipated. To achieve a clinically meaningful effect from DR repair, it was thought the anatomical failure rate should be at least halved to a 20% re-tear rate. Allowing for a 15% standard deviation within groups, it was determined that 20 patients per group would provide sufficient statistical power (80%) to detect a significant difference between the groups (*P* ≤ 0.05) for a re-tear rate.

From January 2007 to October 2008, the senior author performed 182 shoulder arthroscopies for several different pathologies. Inclusion and exclusion criteria for the current study are listed in Table 1. Previous corticosteroid injections in the same shoulder was not an exclusion criterion. Clinical files and intraoperative videos were reviewed to confirm operated side, age of the patients at surgery, date of surgery, associated surgical procedures, associated intra-articular pathologies, surgical time, implant expense, and inclusion and exclusion criteria.

**Table 1:** Inclusion and exclusion criteria

**Inclusion criteria**	**Exclusion criteria**
Full-thickness tears	Less than three years of follow-up
Medium-sized full-thickness tears [39]	Massive retracted tears at surgery
Primary repair	Partial tears at surgery
Degenerative and traumatic tears	Associated subscapularis tendon tears
Long Head Biceps Brachi diseases	Subsequent surgery in the same shoulder
Written informed consent	Preoperative MRI unavailable
	Previous infection in the same shoulder
	Cuff tear arthropathy

Fifty patients met our inclusion and exclusion criteria. These patients were called, and the aims and scopes of the study were explained in details. The patients received additional information about injection of the contrast medium, the risk of infection and the importance of a complete antibiotic prophylaxis. Ten patients were not evaluated at follow-up; reasons for the missed evaluation are listed in Table 2. Forty patients were available for a complete follow-up evaluation, with 20 in the SR group and 20 in the DR group. In all cases, the clinical evaluation was performed first.

**Table 2:** Reasons for the missed follow-up evaluation

**Reason**	**Number of patients**
Unable to locate	3
Too far from the hospital	3
Refused MRA contrast medium injection	2
Good function of the shoulder, do not want further evaluation	1
Poor function of the shoulder, consulted another surgeon	1

Before the clinical and MRA evaluations, all the patients were advised again about the risks and benefits of the procedure and all the patients signed a standard written informed consent. The follow-up protocol was approved by the Institutional Review Board at our Institution.

### Surgical techniques

All the procedures were performed by the senior author, after general endotracheal anaesthesia and insterscalene nerve block, with the patient in the beach chair position. In both groups posterior, anterior and 3 to 4 lateral portals were established for each patient. The posterior portal was used as the viewing portal; the anterior portal and the lateral portals were used as the working portals. Briefly, for SR repair 2 No2 double loaded suture anchors (Arthrex, Naples, Florida, USA) were placed in the greater tuberosity. Sutures were passed through the cuff with a suture passer (Arthrex, Naples, Florida, USA) and tied with a simple knot and a mattress knot for each anchor. DR repair was performed according to the “DR double-pulley technique”, as described by Arrigoni et al. [42]. Briefly, 2 No2 double loaded suture anchors (Arthrex, Naples, Florida, USA) were placed very close to the articular cartilage to form the medial row. Then, wires were passed through the cuff with a suture passer, and wires of one color were tied together with a simple knot and an outside-in knot. One wire of the other color was retrieved from each anchor, placed into a push-lock (Arthrex, Naples, Florida, USA) and fixed to the anterolateral part of the greater tuberosity. The same procedure was repeated with the last two wires that were fixed with a push-lock to the posterolateral aspect of the greater tuberosity. For both SR and DR repair, only one strand of the suture was passed on the tendon at each time so as to avoid creating large holes through the cuff.

### Postoperative protocol

All the patients followed the same postoperative protocol, as described in detail in Table 3. Briefly, they wore a brace 24 h a day with the operated shoulder at 15 degrees of abduction and in neutral rotation. During this early phase, bracing was discontinued only for bathing or taking a shower. Subsequently, a scheduled program of passive physical therapy 2 to 3 times a week was started. Only after a complete passive range of motion had been achieved, active assisted exercises and progressive muscle strengthening were begun. Patients returned to their normal activities of daily living 3 to 6 months after surgery. Sports and recreational activities were allowed 6 to 12 months after surgery.

**Table 3:** Postoperative protocol

**PHASE**	
Phase I	
Days 1 to 14	Brace in abduction at 15°
	Fingers, wrist, elbow and cervical spine movements
Days 15 to 28	Brace in abduction at 15°
	Pendulum exercises
	Passive movement of the shoulder up to 90°, avoiding rotations and pain.
	Hydrotherapy after day 14
Phase II	
Weeks 5 to 12	Progressive brace removal
	Musculoskeletal core strengthening
	Strengthening of scapulothoracic muscles
	Active assisted motion
	Passive movement of the shoulder, including rotations
	Hydrotherapy
	Proprioceptive exercises
Phase III	
Week 13 to 6 months	Muscular strengthening with Thera-Band
	Proprioceptive exercises
Phase IV	
After 6 months	For people who are involved in sports and recreational activities, progressive introduction of sport-specific exercises without pain

### Clinical evaluation

All the patients were followed up 15 days after surgery for stitches removal, and then at 1, 3, 6, 12 and 24 months postoperatively. All the patients were evaluated clinically by the same author at the last follow-up (at least 3 years after surgery). Clinical assessment included a complete physical examination, the CMS [40] and the SST [41]. These are two widely accepted and reliable forms for evaluating the shoulder. Patients were asked about their work activities, retirement status, smoking, drinking, physical and sports activities and comorbidities. Finally, they were also asked to state what was their most important complaint about the operated shoulder during the past year.

### Imaging evaluation

All the patients received 875 mg of Amoxicillin and 125 mg of Clavulanic acid twice a day for 4 days starting the day of the procedure. All the procedures were performed by the same author (who is a trained musculoskeletal radiologist). After thorough disinfection of the skin over the operated shoulder, under ultrasonographic guidance (Hitachi Logos Hi Vision E, Hitachi, Ltd. 1-6-6 Marunouchi, Chiyoda-ku, Tokyo 100–8280, Japan) a 20 G Chiba needle was inserted into the articular cavity just below and lateral to the coracoid process, and 20 ml of Gadolinium solution (Magnevist, Bayer Schering Pharma AG, Berlin, Germany) were injected into the capsule. Magnetic resonance scans were acquired with a 3 Tesla MR scanner (Achieva 3.0 T, Koninklijke Philips Electronics N.V., Eindhoven, the Netherlands). In all patients, MRA scanning was completed within 40 minutes after injection of the contrast medium into the shoulder. The mean scanning time was 15 ± 1.5 minutes.The standard MRA study was composed of T1 Turbo Spin Echo (TSE) sequences on the axial, sagittal and coronal oblique planes (TR: 450 ms; TE 20 ms; 3 mm slice, 1.5 mm gap; matrix: ax 200 × 150; cor 176 × 140; sag 228 × 160) and 3D WATS-C FFE sequence (TR: 20 ms; TE 50 ms; 1.2 mm slice, 0 mm gap; matrix 256 × 256). Re-tears were classified according to Cho et al. [43]: a re-tear at the tendon-to-bone interface was classified as type 1, and a re-tear at the musculotendinous junction was classified as type 2. We decided to consider as re-tear only the full-thickness tears, in order to simplify the evaluation and the comparison with the clinical data. The diagnosis of re-tears was based on the global evaluation of many parameters, such as tendon thickness, retraction, intensity on different sequences, and insertion site.

### Statistical analysis

An unpaired t-test was used to compare objective outcomes assuming unequal variances between groups. Categorical variables were compared using a Chi-square test between both groups. For all statistical tests, the alpha level was set at 0.05. Statistical analyses were performed with SPSS v.15.0 (SPSS Inc., an IBM Company, Chicago, IL, USA).

## Results

Tables 4 and 5 summarize comorbidities, additional surgical procedures and results in the two groups of patients.

**Table 4:** Demographics and clinical data

	**SR group**	**DR group**
Associated surgical procedures		
- LHBB tenotomy		7
- Mumford		1
Comorbidities		
- Diabetes	1	1
- COPD		1
- Thyroid nodules		1
- RCU	1	2
- Hashimoto’s disease	1	
- Sjögren’s disease	1	

**Table 5:** Results at follow-up

	**SR**	**DR**	**P-level for statistical comparison**
Males : Females	13 : 7	12 : 8	0.74
Age (years)	66 ± 8	63 ± 7	0.27
Dominant arm	12	17	0.08
Follow-up (months)	40.0 ± 5.0	38.9 ± 2.3	0.18
Preoperative CMS (points)	45 ± 10	42 ± 12	0.40
Postoperative CMS (points)	70 ± 9	67 ± 15	0.33
Preoperative SST (points)	7.3 ± 1.6	7.8 ± 2.0	0.39
Postoperative SST (points)	9.4 ± 1.7	10.1 ± 2.0	0.28
Surgical time (minutes)	92 ± 13	104 ± 15	0.001
Implant expense (Euros)	400 ± 143	600 ± 138	0.00006
Re-tears	12 (60%)	5 (25%)	0.02

Continuous variables are reported as mean ± standard deviation.

In the SR group there were 13 males and 7 females, the average age at follow-up was 66 ± 8 (range 47 – 78) years, and the dominant arm was involved in 12 cases. The onset of symptoms was traumatic in 6 patients.

In the DR group there were 12 males and 8 females, the average age at follow-up was 63 ± 7 (range 57 – 73) years, and the dominant arm was involved in 17 cases. The onset of symptoms was traumatic in 5 patients.

**Figure 1:**
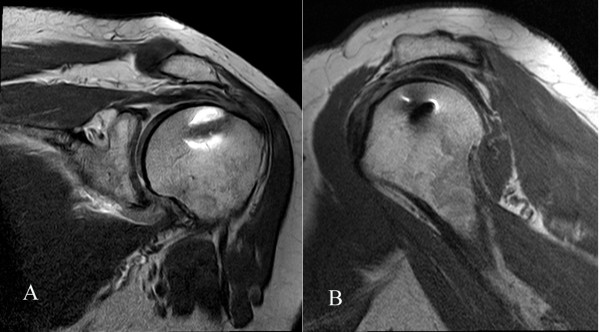
**Coronal (A) and oblique sagittal (B) MRA views of the operated shoulder of a 69-year-old woman (SR group), at 49-month follow-up, showed a continuous supraspinatus tendon 5 mm thick, without muscular retraction.** The humeral head was not superiorly migrated. Clinical results were excellent with 85 points at CMS and 12 points at SST.

In the SR group, the mean preoperative CMS and SST scores were 45 ± 10 (range 38–50) and 7.3 ± 1.6 (range 5–8) points respectively. The mean surgical time was 92 (range 73–118) minutes. The mean implant expense was 400 (range 250–600) Euros. At a mean 40.0 ± 5.0 (range 36 – 50) month follow-up, the mean CMS and the mean SST scores were 70 ± 9 (range 58 – 85) and 9.4 ± 1.7 (range 6 – 12) points respectively. In the SR group, 8 patients showed a good reinsertion of the rotator cuff (Figure 1) while a re-tear was observed in 12 patients (re-tear rate 60%). There were five type I and seven type II re-tears [43].

**Figure 2:**
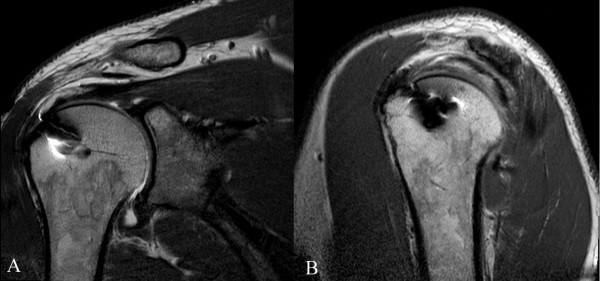
**Coronal (A) and oblique sagittal (B) MRA views of the operated shoulder of a 57-year-old man (DR group), at 37-month follow-up, showed a continuous supraspinatus tendon 8 mm thick, with leakage of the contrast medium at the suture anchor level.** There was no tendon and/or muscle retraction nor superior migration of the humeral head. Clinical results were excellent with 89 points at CMS and 12 points at SST.

In the DR group, the mean preoperative CMS and SST scores were 43 ± 12 (range 24–52) and 7.8 ± 2.0 (range 6–9) points respectively. The mean surgical time was 104 (range 85–136) minutes. The mean implant expense was 600 (range 450–800) Euros. At a mean 38.9 ± 2.3 (range 36 – 43) month follow-up, the mean CMS and the mean SST scores in the DR group were 67 ± 15 (range 37 – 89) and 10.1 ± 2.0 (range 7 – 12) points respectively. In the DR group, 15 patients showed a good reinsertion of the rotator cuff (Figure 2), while a re-tear was observed in 5 patients (re-tear rate 25%) and they were all type II [43].

The group comparisons showed no significant differences in age, gender, side involved, preoperative CMS and SST scores, length of follow-up and comorbidities between the two groups (Table 5).

**Figure 3:**
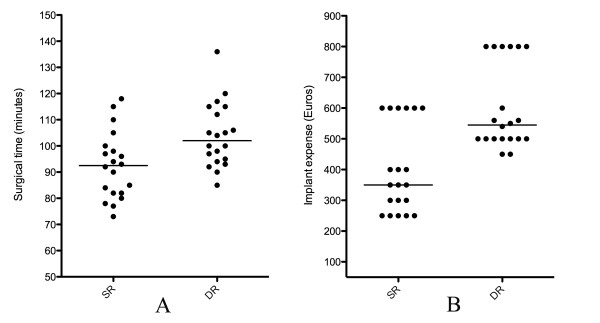
**Vertical dot-plot graphic showing individual values for surgical time (A) and implant expense (B) in SR and DR groups.** Median levels are represented by the horizontal lines.

At follow-up, the CMS and SST scores significantly improved compared to the preoperative values, but there were no statistically significant differences between the two groups in these clinical outcomes. In the DR group there was a statistically significant lower re-tear rate, with longer surgical time and greater implant expense (Table 5). The analysis of data showed a median level of 92,5 and 102 minutes in surgical time and a median level of 350 and 545 Euros in implant expense in SR and DR groups respectively (Figure 3). There were not enough patients for a statistical correlation between re-tear localization and repair technique.

The most frequent complaint at follow-up (75% of the patients in both SR and DR groups) was lack of strength in the operated shoulder during activities of everyday life and/or work activities. Three patients in the SR group and 1 patient in the DR group reported that sometimes they woke up at night owing to pain in the operated shoulder.

The best clinical results were obtained in younger patients, but no definitive statistically significant correlation between age and clinical results could be defined because the two groups were too small for a sub-group analysis. One patient was a boxing trainer, and he used to fight in noncompetitive matches. He returned to his sports activities without any complaint 8 months after surgery. At follow-up, one patient in the SR group showed a mild scapular dyskinesis in the operated shoulder and he was prescribed physical therapy [46]. At a subsequent visit 2 months later, he had completely recovered scapulothoracic function.

## Discussion

We found no clinically significant differences between SR and DR repair groups, but MRA showed a significantly lower re-tear rate in the DR group compared to the SR group (25% vs. 60%. P = 0.02). To the best of our knowledge, this is the first report on a 3 Tesla MRA in the evaluation of two different techniques for rotator cuff repair. A 3 Tesla scanner was used in order to reduce scanning time and improve image quality. The short scanning time (15 ± 1.5 minutes) resulted in great patient satisfaction and low anxiety levels. Moreover, with intra-articular contrast medium, images have to be acquired within 40 minutes from the injection and a short scanning time helps to facilitate examination of the patient.

In previous reports, the use of 3 Tesla MR imaging has been shown to improve knee imaging because the signal-to-noise ratio for cartilage is significantly higher than at 1.5 Tesla [30,36]. The signal-to-noise ratio obtained at 3 Tesla can also be used to obtain higher spatial resolution and/or to reduce the acquisition time. In addition, a recent study showed that, by using higher field strength, MR images of the ankle were obtained with excellent diagnostic quality and a reduction in imaging time of about 44% [28]. Many studies have also been published about evaluation of the shoulder with a 3 Tesla magnet, with and without intra-articular contrast medium (MRA). The increase in signal offered by the high field enables improved visualization of bone, cartilage, tendons and ligaments. Thanks to the greater signal-to-noise ratio and improved spatial resolution, MR imaging at 3 Tesla is able to notably increase diagnostic performance in the musculoskeletal setting, compared to 1.5 Tesla, allowing for an unprecedented level of bone and soft tissue detail, with consequent improvement in patient treatment and management [28-38]. For these reasons, evaluating the rotator cuff in operated patients with a 3 Tesla MRA permits a better imaging quality that improves detection of tears and ruptures. Moreover, assessment of the integrity of other structures is enhanced.

There are few studies in literature directly comparing the clinical results of SR and DR repair in the same setting, and none of them demonstrated any statistically significant difference between the two techniques [20,24-27,44,45]. Our clinical results agree with those previously reported in literature; in fact, the SR and DR groups showed similar results on the CMS and SST, without any statistically significant difference.

To the best of our knowledge, only Franceschi et al. [25] have reported on operative time and implant expense and concluded that DR repair has a statistically significant longer surgical time and a greater implant expense than SR repair. In agreement with Franceschi et al. [25], DR repair in our study entailed longer surgical time and greater implant expense.

There are also few studies directly comparing the radiological results of SR and DR repair in the same setting [4,22-25,45]. The re-tear rates observed in our two groups are similar to those reported in other published studies, ranging from 10% to 90% [3,20-25,43-45]. There are only three level of evidence 1 studies in literature directly comparing the radiographic results of SR and DR rotator cuff repair [24,25,44]. Franceschi et al. [25] in their MRA study on large and massive rotator cuff tears, after 2 years of follow-up, reported 10 partial thickness defects and 2 full-thickness defects in 26 patients treated with SR repair, and 7 partial thickness defects and 1 full-thickness defect in 26 patients treated with DR repair. This difference was considered not statistically significant. They concluded that there are no advantages in using a DR technique to restore the anatomical footprint, and the mechanical advantages reported in many cadaveric studies do not translate into superior clinical performance when compared with the more traditional, less demanding, cheaper and more advantageous technique of SR repair. In their MRI study on small and medium-sized rotator cuff tears, Burks et al. [24] reported the same re-tear rate (10%) in both SR and DR repair groups, but they followed up their patients for only one year. More recently, Koh et al. [44] reported a full-thickness re-tear in 16.7% of the SR group patients and in 26.1% of the DR group patients, without any statistically significant difference. With partial re-tears also included, 62.5% of the patients in the SR group and 30.4% of the patients in the DR group had a re-tear; this difference was statistically significant. In their retrospective study, Cho et al. [43] also reported similar re-tear rates following SR or DR rotator cuff repair at MRI 6 months after surgery, without statistically significant differences. The studies by Burks et al. [24], Franceschi et al. [25] and Koh et al. [44] are level of evidence 1 studies, which are supposed to have the least possible bias, even though Franceschi et al. [25] did not calculate the sample size, and Burks et al. [24] argued that in their study there could be a type II error in finding a true difference between the SR and DR groups, which a larger number of patients might have revealed. The prospective study by Koh et al. [44] was focused mainly on clinical results. MRI was performed only in less than 80% of the enrolled patients (77% in the SR group and 74% in the DR group), with a relevant “dropout” rate. The difference for full-thickness re-tear rates was not statistically significant, but with the given sample size the statistical power obtained was low. With partial re-tears also included, this difference was statistically significant; after Bonferroni correction, it was no longer statistically significant. As observed by the authors themselves, this correction test raises the possibility of a type II error and the results of the study should be interpreted with caution. It is important to consider that the results of rotator cuff repair are reported to decrease over time, and maybe the follow-up in these studies [24,25,43,44] is not long enough to demonstrate a difference in the re-tear rate between the two techniques. To the best of our knowledge, the study by Sugaya et al. [45], even though retrospective, with a 3-year follow-up is the longest follow-up study directly comparing SR and DR repairs in the same setting. They reported a 56% re-tear rate in the SR group and a 27% re-tear rate in the DR group at MRI, and the difference was considered statistically significant (p < 0.01). In literature, conflicting results are reported about re-tear rates after SR and DR rotator cuff repair, with high level of evidence studies [8,14] suggesting no differences between the two techniques, and retrospective studies reporting lower re-tear rates for DR repair [9,22,45]. We think that more level of evidence 1 studies, with longer follow-up, are needed to demonstrate whether a difference exists in re-tear rates between SR and DR repair.

Some studies [47,48] reported that the medial side of an intact supraspinatus tendon has equal, or maybe better, biomechanical properties than the lateral side, in terms of stiffness, pullout and work, and a tear in the tendon alters these properties only in the lateral side, but not in the medial side. The better biomechanical properties of the medial side of a torn supraspinatus tendon are related to larger collagen fibrils and greater fibril density, compared to the lateral tendon, that may provide a more robust matrix for resisting suture migration [48]. In contrast, another study reported on similar fixation strength for SR and DR repair [14]. We think that the lower re-tear rate observed in our patients in the DR group may be related to the protective effect of the medial row on the lateral row (strain shielding effect). As observed by Cho et al. [43], this phenomenon may explain why, after SR repair, a re-tear occurs more frequently at the tendon-to-bone interface and, on the other hand, after DR repair, a re-tear occurs more frequently at the musculotendinous junction, because the lateral row is protected by the medial row. In our study, there were too few patients for a statistical correlation between re-tear localization and repair technique.

**Figure 4:**
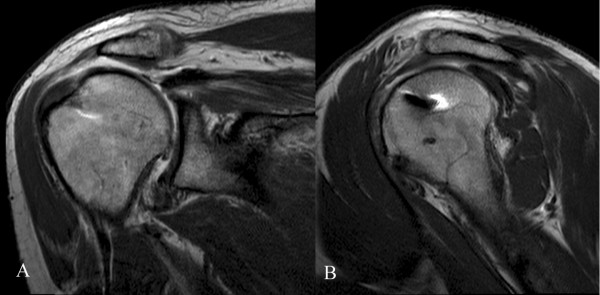
**Coronal (A) and oblique sagittal (B) MRA views of the operated shoulder of a 48-year-old man (SR group), at 38-month follow-up, showed a type II tear of the rotator cuff, with a thin supraspinatus tendon (4.5 mm thick), leakage of the contrast medium, and superior migration of the humeral head.** Despite the radiological findings, clinical results were good with 74 points at CMS and 12 points at SST.

Several studies have documented better subjective and objective results of rotator cuff repair when the tendon has been documented to heal [1,22,45], but we found no statistically significant differences in the clinical scores between our two groups, albeit the re-tear rate was significantly lower in our DR group (Figure 4).

Better subjective and objective results have also been described in younger patients [49,50]. We strongly agree with this observation, even though the small number of young patients in our two groups did not allow us to demonstrate any statistically significant correlation.

We observed some leakage of the contrast medium in all our cases, despite the clinical outcomes, even though in some cases it was very mild, and the tendon appeared healthy (homogeneous with normal thickness and no retraction). We hypothesized that leakage of the contrast medium took place at the interval between insertion of the tendons at the suture anchors level. In the study by Charousset et al. [20], the patients were evaluated with CT arthrography, and the percentage of watertight healed rotator cuffs (no leakage of the contrast medium into the subacromial bursa, indicating perfect healing or a partial thickness defect) was 77.4% for the DR group, compared with 60.0% for the SR group (p > 0.05). In the study of Franceschi et al. [25], the patients were evaluated with MRA, and the percentage of intact cuffs and partial-thickness defects was 96% for the DR group, compared with 92% for the SR group (p > 0.05). Charousset et al. [20] clearly stated that leakage of the contrast medium was not related to anatomic healing with reestablishment of the native footprint. In their study it was achieved in 61.3% of the DR repairs, compared with 40% of the SR repairs, and this difference was significant (p = 0.03). Franceschi et al. [25] in their study did not discuss the difference between leakage of the contrast medium and healing of the footprint. As previously reported by Duc et al. [29], sometimes something that seems a defect is actually an intact tendon, but distorted by a scar. In some cases, patients operated on for a rotator cuff tear, after a period of wellness, can experience pain in the operated shoulder for several reasons, not always related to the shoulder itself. Usually patients ask for, or another physician prescribes, imaging studies, that in some cases are performed with intra-articular contrast medium and/or with an ultrahigh magnetic field (3 Tesla MRA), as suggested by many papers. In such a case the radiologist might interpret the leakage of the contrast medium as a re-tear, without considering other parameters such as tendon thickness, retraction, signal intensity, insertion site, etc., and without any consideration of clinical function. We think that leakage of the contrast medium is due to an incomplete tendon-to-bone sealing, especially near suture anchors, or to an interstitial passage of the liquid among the fibrillated tendon fibers, especially if multiple strands of sutures are passed in the pulley technique through the cuff, creating large holes. We believe that imaging must be prescribed and interpreted on the basis of clinical findings, and leakage of the contrast medium does not in itself mean re-tear. This final consideration could have important medicolegal implications.

In a recent review of the literature about the clinical results of rotator cuff repair, Saridakis et al. [9] suggested that surgeons should use a DR technique only for tears larger than 3 cm. On the other hand, Duquin et al. [51], in a recent review of the literature about radiographic results of rotator cuff repair, including the studies by Burks et al. [24] and Franceschi et al. [25], suggested that surgeons should use a DR technique for all rotator cuff repairs, when possible, particularly for tears greater than 1 cm [51]. On the basis of our clinical findings and MRA results, and in accordance with these reviews [9,51], we now reserve DR rotator cuff repairs for more active patients, with larger tear sizes, and SR repair for older, less active patients and for patients with smaller tear sizes.

Significant limitations are present in our study. First of all, this is a retrospective study with a relatively medium-term follow-up, and the radiologist was not blinded to the repair technique. But we have to consider that, in every study setting, even prospective ones, which compares the radiographic results of SR and DR repair, the radiologist cannot be really blinded about the surgical technique. In fact, he/she can count on MRA/MRI/CT scans the number of suture anchors placed in the footprint and determine whether they are in a SR or DR fashion. This introduces a potential, significant bias in the results reported by the radiologist even in prospective and “well-done” studies, which cannot be avoided. Furthermore, in our study the patients were not evaluated at a fixed follow-up time, but it ranged from 36 to 50 months in the SR group and from 36 to 43 months in the DR group. However, there was not a statistically significant difference in the mean length of follow-up between the two groups.

Our study also has some strengths. It is a single-surgeon series, with uniform surgical skills; we adopted rigid inclusion and exclusion criteria. The “dropout” rate in our study (20%) is acceptable and within the limits for “high-quality studies”. Injection of the contrast medium was done under ultrasound control to confirm intra-articular release, and all the patients were evaluated with a 3 Tesla MRA, which is able to notably increase diagnostic performance in the musculoskeletal setting, allowing for an unprecedented level of bone and soft tissue detail, with consequent improvement in patient treatment and management [28-38].

## Conclusion

To the best of our knowledge, this is the first report on a 3 Tesla MRA in the evaluation of two different techniques for rotator cuff repair. DR repair resulted in a statistically significant lower re-tear rate, despite no difference in clinical outcomes. We think that leakage of the contrast medium is due to an incomplete tendon-to-bone sealing, which is not a re-tear. This phenomenon could have important medicolegal implications.

## Abbreviations

CMS: Constant-murley scale; DR: Double row; MRA: Magnetic resonance arthrography; SR: Single row; SST: Simple shoulder test.

## Competing interests

The authors report no conflict of interest.

## Authors’ contributions

CT: has made substantial contributions to conception and design of the clinical data, as well as critical revision of the manuscript, and has given final approval of the version to be published. SB: has made substantial contributions to the acquisition, analysis and interpretation of clinical data and has been involved in drafting the manuscript. ES: has made substantial contributions to conception and design and has been involved in drafting the manuscript. RF: has made substantial contributions to the acquisition, analysis and interpretation of radiological data. DAB: has made substantial contributions to the acquisition of radiological data and has been involved in drafting the manuscript. SM: has made substantial contributions to conception and design of the radiological data and has given final approval of the version to be published. GS: has made substantial contributions to conception and design of the radiological data and has given final approval of the version to be published. All authors read and approved the final manuscript.

## Pre-publication history

The pre-publication history for this paper can be accessed here:

http://www.biomedcentral.com/1471-2474/14/43/prepub

## References

[B1] Bishop J Klepps S Lo IK Bird J Gladstone JN Flatow EL Cuff integrity after arthroscopic versus open rotator cuff repair: a prospective study J Shoulder Elbow Surg 2006 15 290 299 10.1016/j.jse.2005.09.017 16679227

[B2] Kang L Henn RF Tashjian RZ Green A Early outcome of arthroscopic rotator cuff repair: a matched comparison with mini-open rotator cuff repair Arthroscopy 2007 23 573 582 e1-2 10.1016/j.arthro.2007.01.011 17560471

[B3] Nho SJ Shindle MK Sherman SL Freedman KB Lyman S MacGil-livray JD Systematic review of arthroscopic rotator cuff repair and mini-open rotator cuff repair J Bone Joint Surg Am 2007 89 Suppl 3 127 136 17908878 10.2106/JBJS.G.00583

[B4] Kasten P Keil C Grieser T Raiss P Streich N Loew M Prospective randomised comparison of arthroscopic versus mini-open rotator cuff repair of the supraspinatus tendon Int Orthop 2011 35 1663 1670 10.1007/s00264-011-1262-2 21533643 PMC3193969

[B5] Ropiak RR Zmistowski BM Ciccotti MC Rynning R Williams GR Jr Fenlin JM Jr Postoperative Pain after Open versus Arthroscopic Rotator Cuff Repair 2010 Scottsdale, AZ: ASES 2010 closed meeting

[B6] Ma CB Comerford L Wilson J Puttlitz CM Biomechanical evaluation of arthroscopic rotator cuff repairs: double-row compared with single-row fixation J Bone Joint Surg Am 2006 88 403 410 10.2106/JBJS.D.02887 16452754

[B7] Meier SW Meier JD The effect of double-row fixation on initial repair strength in rotator cuff repair: a biomechanical study Arthroscopy 2006 22 493 497 10.1016/j.arthro.2006.07.004 17084292

[B8] Milano G Grasso A Zarelli D Deriu L Cillo M Fabbriciani C Comparison between single-row and double-row rotator cuff repair: a biomechanical study Knee Surg Sports Traumatol Arthrosc 2008 16 75 80 10.1007/s00167-007-0382-0 17684730

[B9] Saridakis P Jones G Outcomes of single-row and double-row arthroscopic rotator cuff repair: a systematic review J Bone Joint Surg Am 2010 92 732 742 10.2106/JBJS.I.01295 20194334

[B10] Smith CD Alexander S Hill AM Huijsmans PE Bull AM Amis AA De Beer JF Wallace AL A biomechanical comparison of single and double-row fixation in arthroscopic rotator cuff repair J Bone Joint Surg Am 2006 88 2425 2431 10.2106/JBJS.E.00697 17079400

[B11] Waltrip RL Zheng N Dugas JR Andrews JR Rotator cuff repair. A biomechanical comparison of three techniques Am J Sports Med 2003 31 493 497 12860534 10.1177/03635465030310040301

[B12] Apreleva M Ozbaydar M Fitzgibbons PG Warner JJ Rotator cuff tears: the effect of the reconstruction method on three-dimensional repair site area Arthroscopy 2002 18 519 526 10.1053/jars.2002.32930 11987064

[B13] Brady PC Arrigoni P Burkhart SS Evaluation of residual rotator cuff defects after in vivo single- versus double-row rotator cuff repairs Arthroscopy 2006 22 1070 1075 10.1016/j.arthro.2006.05.007 17027404

[B14] Mazzocca AD Millett PJ Guanche CA Santangelo SA Arciero RA Arthroscopic single-row versus double-row suture anchor rotator cuff repair Am J Sports Med 2005 33 1861 1868 10.1177/0363546505279575 16210578

[B15] Nelson CO Sileo MJ Grossman MG Serra-Hsu F Single-row modified mason-allen versus double-row arthroscopic rotator cuff repair: a biomechanical and surface area comparison Arthroscopy 2008 24 941 948 10.1016/j.arthro.2008.03.011 18657744

[B16] Park MC ElAttrache NS Tibone JE Ahmad CS Jun BJ Lee TQ Part I: Footprint contact characteristics for a transosseous-equivalent rotator cuff repair technique compared with a double-row repair technique J Shoulder Elbow Surg 2007 16 461 468 10.1016/j.jse.2006.09.010 17321161

[B17] Tuoheti Y Itoi E Yamamoto N Seki N Abe H Minagawa H Okada K Shimada Y Contact area, contact pressure, and pressure patterns of the tendon-bone interface after rotator cuff repair Am J Sports Med 2005 33 1869 1874 10.1177/0363546505278256 16157853

[B18] Ahmad CS Stewart AM Izquierdo R Bigliani LU Tendon-bone interface motion in transosseous suture and suture anchor rotator cuff repair techniques Am J Sports Med 2005 33 1667 1671 10.1177/0363546505278252 16093532

[B19] Park MC Cadet ER Levine WN Bigliani LU Ahmad CS Tendon-to-bone pressure distributions at a repaired rotator cuff footprint using transosseous suture and suture anchor fixation techniques Am J Sports Med 2005 33 1154 1159 10.1177/0363546504273053 16000662

[B20] Charousset C Grimberg J Duranthon LD Bellaiche L Petrover D Can a double row anchorage technique improve tendon healing in arthroscopic rotator cuff repair? A prospective, nonrandomized, comparative study of double-row and single-row anchorage techniques with computed tomographic arthrography tendon healing assessment Am J Sports Med 2007 35 1247 1253 10.1177/0363546507301661 17452513

[B21] Galatz LM Ball CM Teefey SA Middleton WD Yamaguchi K The outcome and repair integrity of completely arthroscopically repaired large and massive rotator cuff tears J Bone Joint Surg Am 2004 86 219 224 14960664 10.2106/00004623-200402000-00002

[B22] Lafosse L Brozska R Toussaint B Gobezie R The outcome and structural integrity of arthroscopic rotator cuff repair with use of the double-row suture anchor technique J Bone Joint Surg Am 2007 89 1533 1541 10.2106/JBJS.F.00305 17606793

[B23] Pennington WT Gibbons DJ Bartz BA Dodd M Daun J Klinger J Popovich M Butler B Comparative analysis of single-row versus double-row repair of rotator cuff tears Arthroscopy 2010 26 1419 1426 10.1016/j.arthro.2010.03.013 20875720

[B24] Burks RT Crim J Brown N Fink B Greis PE A prospective randomized clinical trial comparing arthroscopic single- and double-row rotator cuff repair: magnetic resonance imaging and early clinical evaluation Am J Sports Med 2009 37 674 682 10.1177/0363546508328115 19204365

[B25] Franceschi F Ruzzini L Longo UG Martina FM Zobel BB Maffulli N Denaro V Equivalent clinical results of arthroscopic single-row and double-row suture anchor repair for rotator cuff tears: a randomized controlled trial Am J Sports Med 2007 35 1254 1260 10.1177/0363546507302218 17554104

[B26] Grasso A Milano G Salvatore M Falcone G Deriu L Fabbriciani C Single-row versus double-row arthroscopic rotator cuff repair: a prospective randomized clinical study Arthroscopy 2009 25 4 12 10.1016/j.arthro.2008.09.018 19111212

[B27] Park JY Lhee SH Choi JH Park HK Yu JW Seo JB Comparison of the clinical outcomes of single- and double-row repairs in rotator cuff tears Am J Sports Med 2008 36 1310 1316 10.1177/0363546508315039 18413680

[B28] Bauer JS Banerjee S Henning TD Krug R Majumdar S Link TM Fast high-spatial-resolution MRI of the ankle with parallel imaging using GRAPPA at 3 T AJR Am J Roentgenol 2007 189 240 245 10.2214/AJR.07.2066 17579177

[B29] Duc SR Mengiardi B Pfirrmann CW Jost B Hodler J Zanetti M Diagnostic performance of MR arthrography after rotator cuff repair AJR Am J Roentgenol 2006 186 237 241 10.2214/AJR.04.1818 16357409

[B30] Gold GE Han E Stainsby J Wright G Brittain J Beaulieu C Musculoskeletal MRI at 3.0 T: relaxation times and image contrast AJR Am J Roentgenol 2004 183 343 351 15269023 10.2214/ajr.183.2.1830343

[B31] Jung JY Yoon YC Choi SH Kwon JW Yoo J Choe BK Three-dimensional isotropic shoulder MR arthrography: comparison with two-dimensional MR arthrography for the diagnosis of labral lesions at 3.0 T Radiology 2009 250 498 505 10.1148/radiol.2493071548 19188318

[B32] Lambert A Loffroy R Guiu B Mejean N Lerais JM Cercueil JP Krausé D Rotator cuff tears: value of 3.0T MRI J Radiol 2009 90 583 588 10.1016/S0221-0363(09)74024-7 19503046

[B33] Magee T 3-T MRI of the shoulder: is MR arthrography necessary? AJR Am J Roentgenol 2009 192 86 92 10.2214/AJR.08.1097 19098184

[B34] Magee T Williams D 3.0-T MRI of the supraspinatus tendon AJR Am J Roentgenol 2006 187 881 886 10.2214/AJR.05.1047 16985129

[B35] Major NM Browne J Domzalski T Cothran RL Helms CA Evaluation of the glenoid labrum with 3-T MRI: is intraarticular contrast necessary? AJR Am J Roentgenol 2011 196 1139 1144 10.2214/AJR.08.1734 21512082

[B36] Masi JN Sell CA Phan C Han E Newitt D Steinbach L Majumdar S Link TM Cartilage MR imaging at 3.0 versus that at 1.5 T: preliminary results in a porcine model Radiology 2005 236 140 150 10.1148/radiol.2361040747 15987970

[B37] Murray PJ Shaffer BS Clinical update: MR imaging of the shoulder Sports Med Arthrosc 2009 17 40 48 10.1097/JSA.0b013e31819602a6 19204551

[B38] Trattnig S Mamisch TC Noebauer I High-field and ultrahigh-field magnetic resonance imaging: new possibilities for imaging joints Z Rheumatol 2006 65 681 687 10.1007/s00393-006-0121-9 17106667

[B39] DeOrio JK Cofield RH Results of a second attempt at surgical repair of a failed initial rotator cuff repair J Bone Joint Surg Am 1984 66 563 567 6707035

[B40] Constant CR Murley AH A clinical method of functional assessment of the shoulder Clin Orthop Relat Res 1987 214 160 164 3791738

[B41] Matsen FA III Lippitt SB Sidles JA Harryman DT II Practical Evaluation and Management of the Shoulder 1996 Philadelphia: Saunders C

[B42] Arrigoni P Brady PC Burkhart SS The double-pulley technique for double-row rotator cuff repair Arthroscopy 2007 23 675 e1-4 17560485 10.1016/j.arthro.2006.08.016

[B43] Cho NS Yi JW Lee BG Rhee YG Retear patterns after arthroscopic rotator cuff repair: single-row versus suture bridge technique Am J Sports Med 2010 38 664 671 10.1177/0363546509350081 20040768

[B44] Koh KH Kang KC Lim TK Shon MS Yoo JC Prospective randomized clinical trial of single- versus double-row suture anchor repair in 2- to 4-cm rotator cuff tears: clinical and magnetic resonance imaging results Arthroscopy 2011 27 453 462 10.1016/j.arthro.2010.11.059 21444007

[B45] Sugaya H Maeda K Matsuki K Moriishi J Functional and structural outcome after arthroscopic full-thickness rotator cuff repair: single-row versus dual-row fixation Arthroscopy 2005 21 1307 1316 10.1016/j.arthro.2005.08.011 16325080

[B46] Burkhart SS Morgan CD Kibler WB The disabled throwing shoulder: spectrum of pathology Part III: The SICK scapula, scapular dyskinesis, the kinetic chain, and rehabilitation Arthroscopy 2003 19 641 661 10.1016/S0749-8063(03)00389-X 12861203

[B47] Lake SP Miller KS Elliott DM Soslowsky LJ Effect of fiber distribution and realignment on the nonlinear and inhomogeneous mechanical properties of human supraspinatus tendon under longitudinal tensile loading J Orthop Res 2009 27 1596 1602 10.1002/jor.20938 19544524 PMC2813200

[B48] Wang VM Wang FC McNickle AG Friel NA Yanke AB Chubinskaya S Romeo AA Verma NN Cole BJ Medial versus lateral supraspinatus tendon properties: implications for double-row rotator cuff repair Am J Sports Med 2010 38 2456 2463 10.1177/0363546510376817 20929937 PMC3772634

[B49] Oh JH Kim SH Kang JY Oh CH Gong HS Effect of age on functional and structural outcome after rotator cuff repair Am J Sports Med 2010 38 672 678 10.1177/0363546509352460 20357401

[B50] Tashjian RZ Hollins AM Kim HM Teefey SA Middleton WD Steger-May K Galatz LM Yamaguchi K Factors affecting healing rates after arthroscopic double-row rotator cuff repair Am J Sports Med 2010 38 2435 2442 10.1177/0363546510382835 21030564

[B51] Duquin TR Buyea C Bisson LJ Which method of rotator cuff repair leads to the highest rate of structural healing? A systematic review Am J Sports Med 2010 38 835 841 10.1177/0363546509359679 20357403

